# Central retinal artery occlusion as a presenting symptom in Eales’ disease: a case report

**DOI:** 10.1186/s13256-023-04003-y

**Published:** 2023-07-06

**Authors:** Dua Masarwa, Eyal Raskin, Keren Haas, Reut Singer, David Hauser

**Affiliations:** 1 Department of Ophthalmology, Barzilai University Medical Center, Ashkelon, Israel; 2 Goldschleger Eye Institute, Sheba Medical Center, Ramat Gan, Israel

**Keywords:** Eales’ disease, CRAO, CRVO, Vasculitis, Arteritis

## Abstract

**Background:**

Eales’ disease is an idiopathic peripheral retinal vasculopathy characterized by retinal phlebitis, ischemia, retinal neovascularization, and recurrent vitreous hemorrhages. But CRAO is an unusual presentation.

**Case presentation:**

A 27-year-old healthy female nurse of Indian descent presented with sudden vision loss in her right eye upon awakening. Central retinal artery occlusion (CRAO), combined with mild central retinal vein occlusion (CRVO), was diagnosed. During the second of three consecutive sessions of hyperbaric oxygen treatments, her vision rapidly improved. One week later, she developed peripheral phlebitis in the same eye. Infectious, inflammatory, and hematologic etiologies were excluded. The systemic evaluation was normal except for a positive Mantoux tuberculin skin test. Following systemic steroidal treatment, she experienced gradual improvement of her vasculitis. Two weeks later, mild retinal phlebitis appeared in her left eye. Eales’ disease was diagnosed after the exclusion of other diseases.

**Conclusion:**

This is an unusual Eales’ disease case, which presented as combined CRAO with mild CRVO. The association of CRAO and Eales’ disease is reported here for the first time, to our best knowledge.

## Background

Eales’ disease is an idiopathic peripheral retinal vasculopathy characterized by vasculitis, ischemia, retinal neovascularization, and recurrent vitreous hemorrhages [1]. Eales’ disease is a diagnosis of exclusion. Peripheral retinal inflammation and recurrent vitreous hemorrhages without other systemic conditions are defining features [2].

## Case presentation

We present a 27-year-old healthy female nurse of Indian descent who presented to the emergency room shortly upon awakening in the morning with right eye vision loss, and no other neurological complaints. The patient’s past medical, ophthalmic, and familial history was unremarkable.

**Fig. 1:**
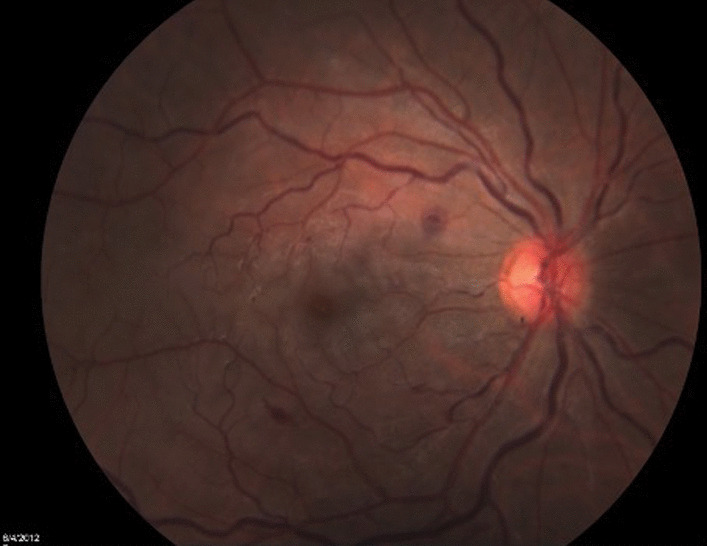
Fundus color picture of the right eye at presentation, showing a mild hyperemic disc, a pale posterior pole, edema temporal to the macula, a few intraretinal hemorrhages, and mildly engorged retinal veins

**Fig. 2:**
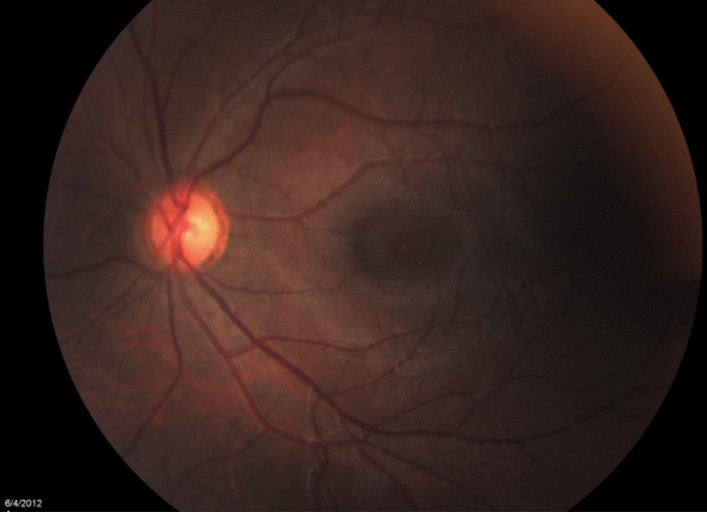
Fundus color picture of the left eye: normal

At presentation, visual acuity (VA) was counting figures for the right eye and 20/20 for the fellow eye, with a positive relative afferent pupillary defect. Anterior segment and intraocular pressure were unremarkable in both eyes. Fundus examination of the right eye revealed a mild hyperemic disc, a pale posterior pole, edema temporal to the macula, a few intraretinal hemorrhages, and mildly engorged retinal veins, with no peripheral retina findings (Fig. 1). Fundus examination of the left eye was unremarkable (Fig. 2).

**Fig. 3:**
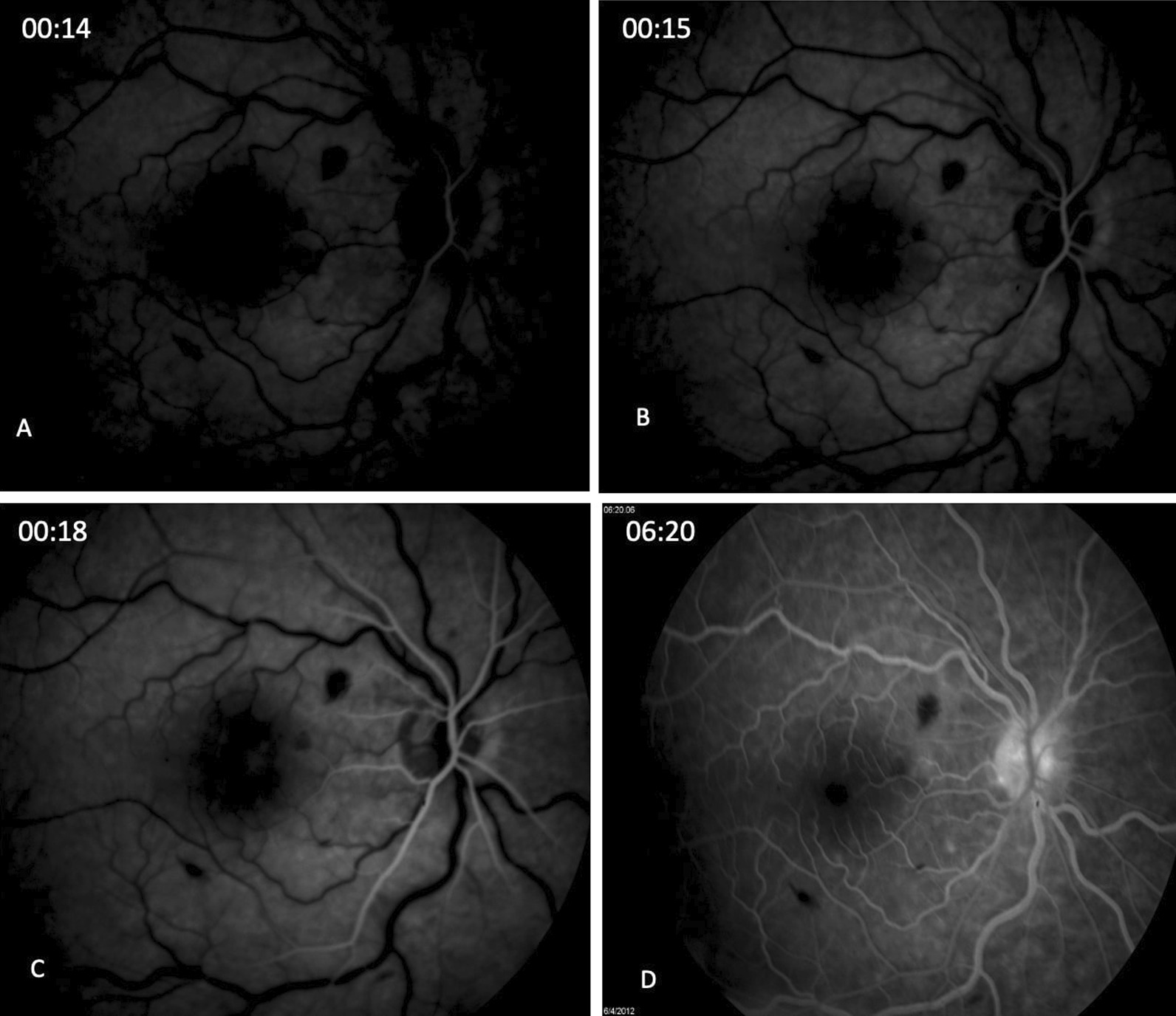
Fluorescein angiography of the right eye. **A, B** at 00:14 and 00:15 seconds, respectively. A significant delay is shown in the arterial filling (**C**) at 00:18 seconds. A delay in the venous filling (**D**) 6:20 seconds shows a “hot” disc in late stages

The patient was referred immediately for fluorescein angiography (FA) test, which revealed a significant delay in the arterial filling, lasting 13 seconds in the right eye; a delay in the venous filling; and a mild “hot” disc in late stages (Fig. 3). She was diagnosed with central retinal artery occlusion (CRAO) combined with mild central retinal vein occlusion (CRVO).

**Fig. 4:**
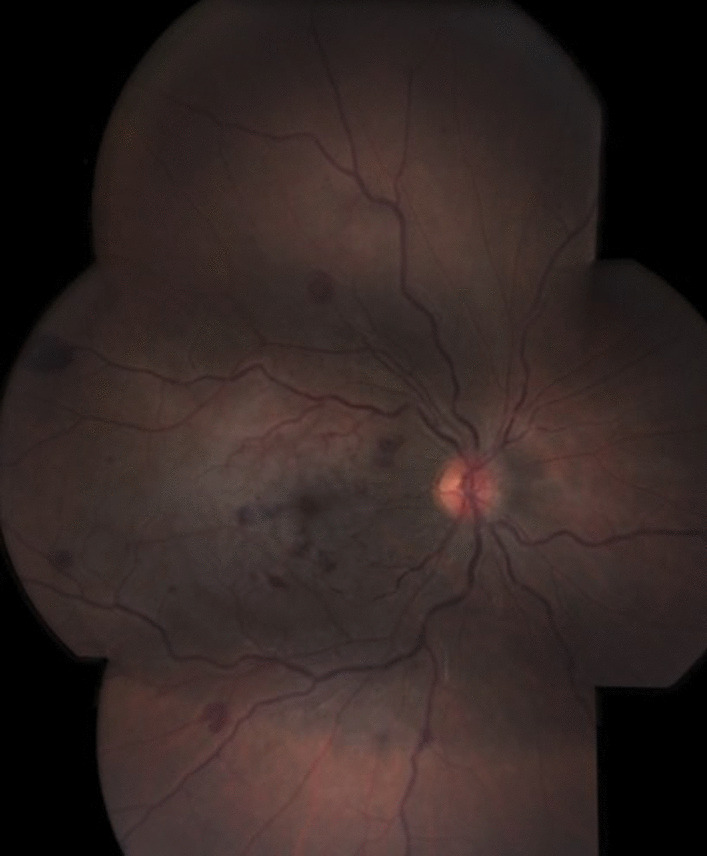
Fundus color picture of the right eye following hyperbaric treatment. More intraretinal hemorrhages are seen

**Fig. 5:**
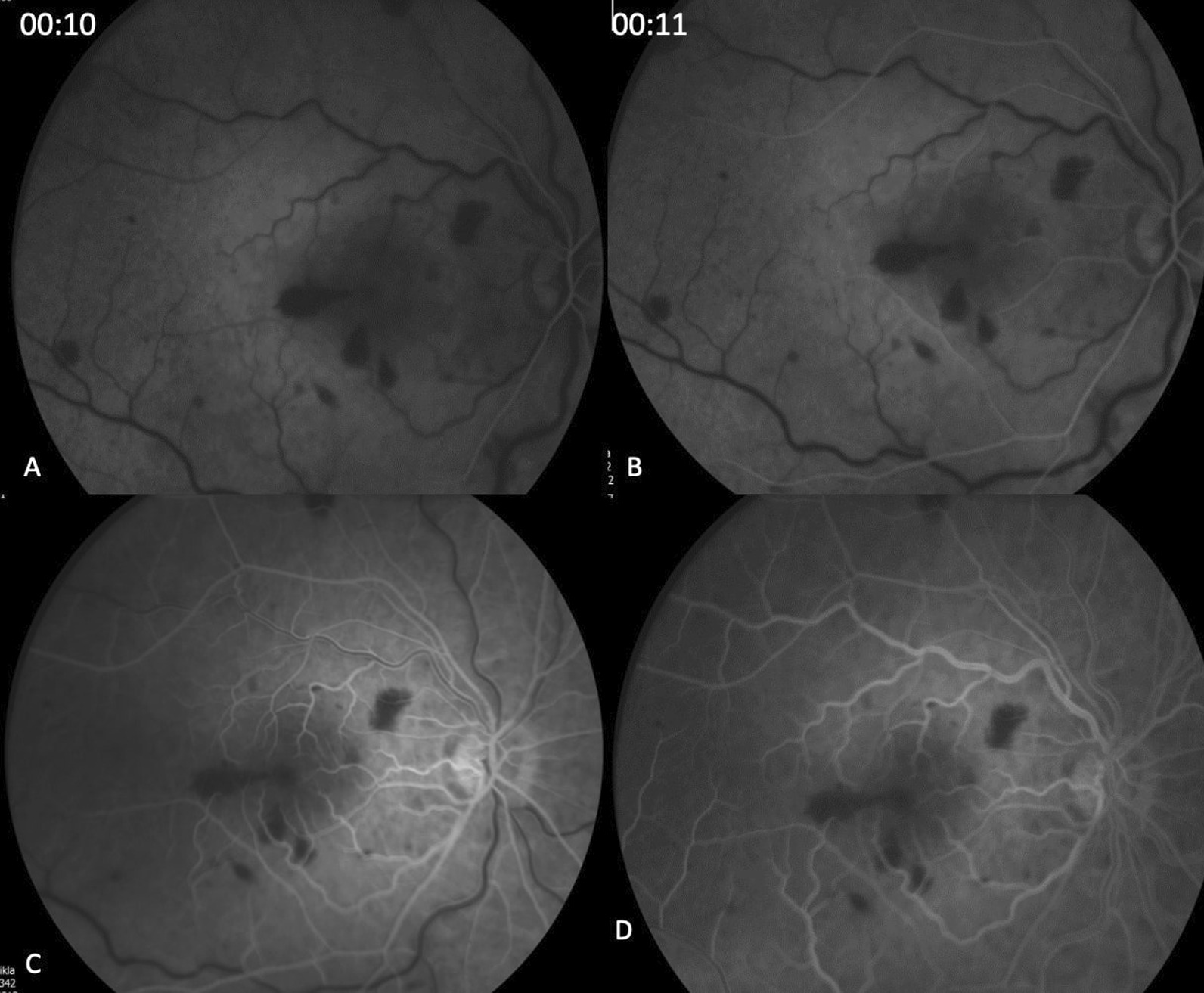
Repeated Fluorescein angiography of the right eye after hyperbaric treatment showing significant improvement in arterial and venous filling time, with remnant delay (**A, B**) at 10 and 11 seconds, respectively; (**C**) at 14 seconds, venous laminar flow; and at (**D**) 19 seconds, complete arterial and venous filling and retinal hemorrhages

**Fig. 6:**
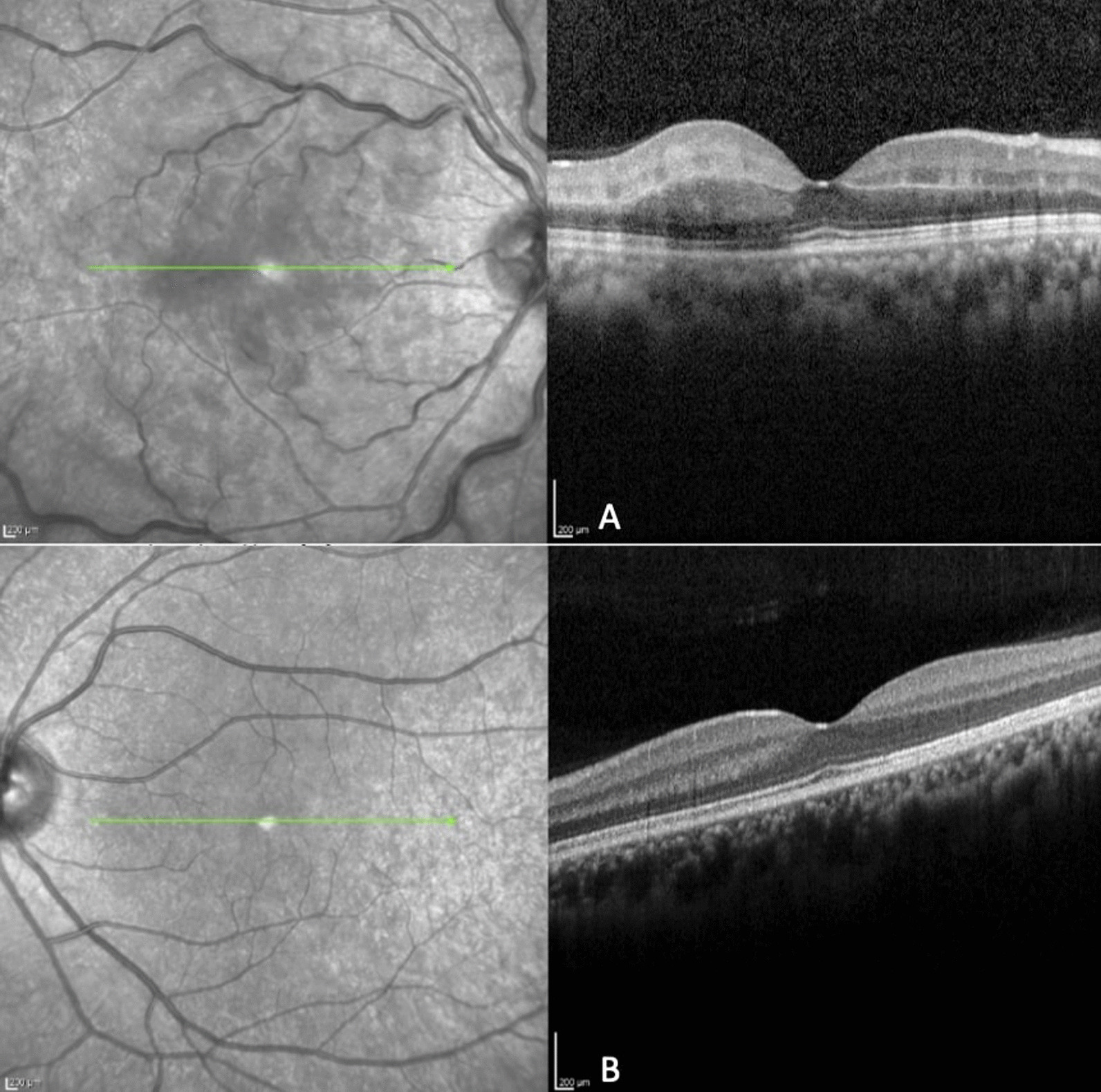
**A** Optical coherence tomography (OCT) of the right eye, showing hyperreflectivity of the inner retinal layers due to retinal edema. **B** OCT of the left eye: unremarkable

On admission, To promote retinal perfusion and improve visual outcomes, a paracentesis was performed on the affected right eye. This procedure leads to a rapid reduction of intraocular pressure, which is particularly beneficial in the first few hours following CRAO [3, 4]; she has immediately referred afterward to a hyperbaric chamber in another hospital. She underwent a series of 3 consecutive sessions of hyperbaric oxygen treatments over 24 hours; The therapy was initiated within less than 20 hours from the onset of her symptoms, which falls within the acceptable time frame for this treatment [5]. The patient recalled rapid and substantial improvement in visual function during the second hyperbaric treatment. The VA improved to 20/40 in the right eye. Fundus examination revealed slightly more intraretinal hemorrhages (Fig. 4). A second FA showed marked improvement in arterial filling time, with remnant delay on arterial and venous filling. (About 5 seconds for the completion of the arterial phase—Fig. 5). Optical coherence tomography showed macular edema in the right eye and a normal fellow eye (Fig. 6). She completed a systemic evaluation, including complete blood count, chemistry, hypercoagulable evaluation, echocardiogram, and carotid Doppler, which were all normal.

**Fig. 7:**
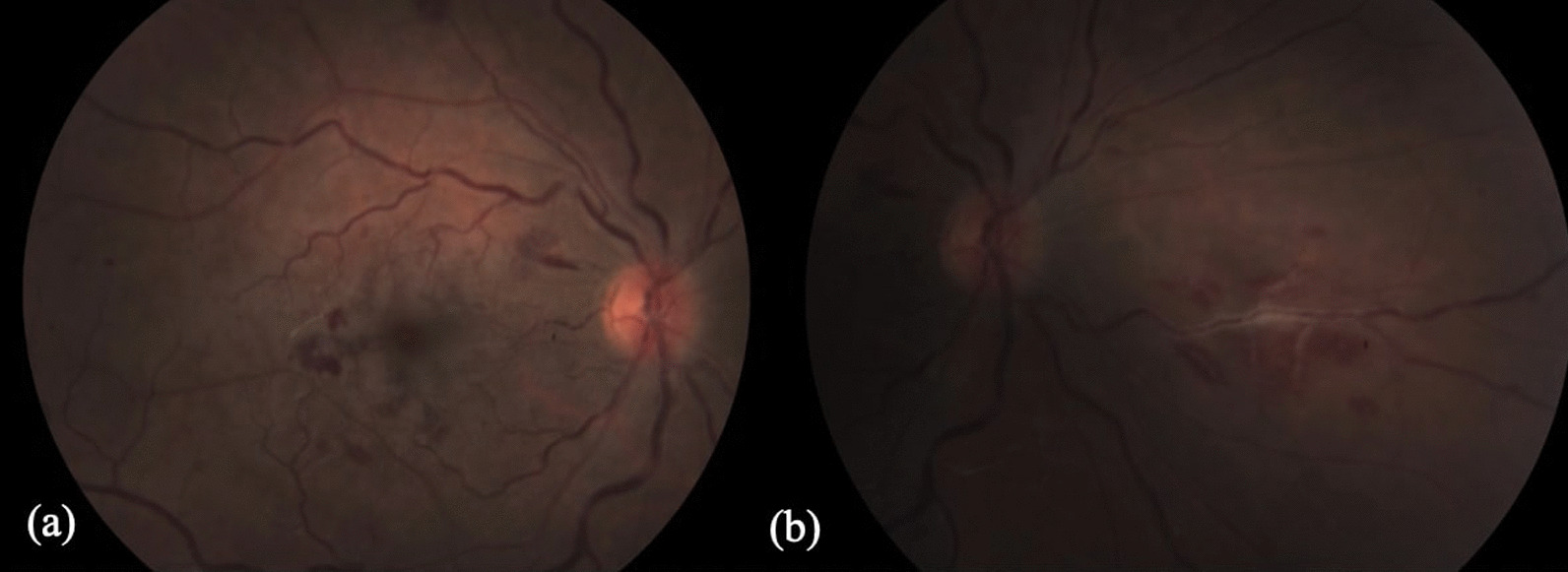
Fundus color pictures of the right eye, one week after the initial presentation. **a** Intraretinal hemorrhages. **b** One focus of phlebitis nasally to the optic disc

**Fig. 8:**
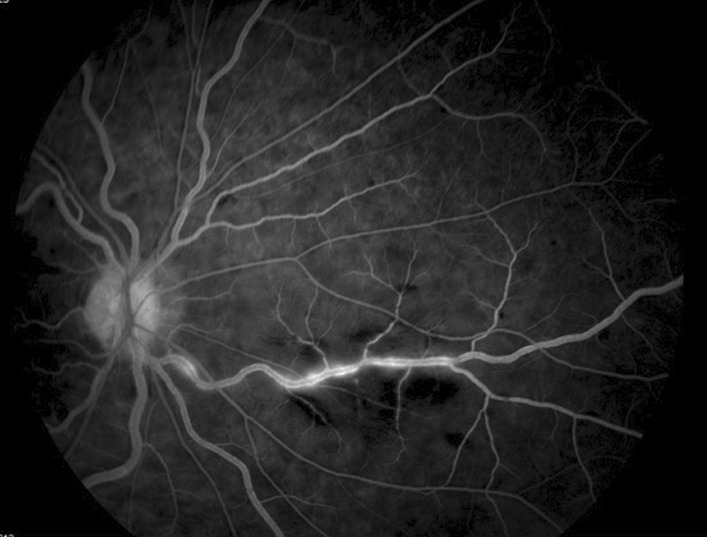
Fluorescein angiography of the right eye at 01:58 seconds, showing late leakage from the inferior retinal artery and blood vessel wall staining

At a one-week follow-up examination, VA remained 20/40 in the right eye. The fundus examination of the right eye showed one focus of phlebitis, nasally to the optic disc (Fig. 7). FA revealed staining if vascular wall and late leakage (Fig. 8), which is consistent with vasculitis. The patient was admitted for reevaluation. Infectious, inflammatory, and hematologic etiologies were excluded, except for the Mantoux tuberculin skin test, which was positive (13 mm); this could indicate previous exposure as a healthcare worker.

**Fig. 9:**
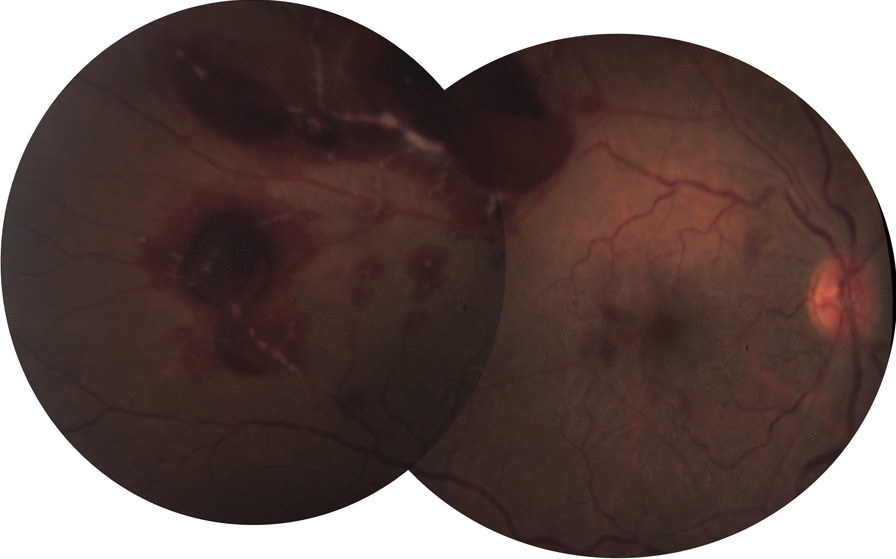
Fundus color picture of the right eye showing vasculitis with retinal hemorrhages close to the macula

During the following two days, VA decreased to 20/125 in the right eye, and the vasculitis spread to the posterior pole, with retinal hemorrhages close to the macula (Fig. 9) Systemic steroidal treatment of intravenous Solumedrol 1 g\day was initiated to control the vasculitis. The VA of the right eye gradually improved to 20/32, without any new vasculitis foci. On the fifth treatment day, new foci of vasculitis appeared on the upper and lower arcades, and a large subretinal hemorrhage at the superior temporal mid-periphery in the same eye. Up to this stage, the fellow eye was not involved.

One week later, small peripheral foci of vasculitis appeared in the left eye for the first time. VA was 20/20 in the left eye. Later, the patient developed peripheral retinal neovascularization in the right eye, treated with sectoral scatter laser photocoagulation for the capillary nonperfusion. The patient was followed closely, in which visits were extended to 6 and then to 12 monthly intervals to date.

She has been followed for over a decade and has remained stable with no disease activity or recurrence.

## Discussion and conclusions

Retinal vasculitis is a sight-threatening inflammation of the retinal vessels. It presents as a painless decrease in vision and is characterized by exudates around the affected vessels, thus resulting in a white sheathing. The diagnosis is confirmed by demonstrating leakage and blood vessel wall staining on fundus FA. Retinal vasculitis can be an isolated condition or a complication of local or systemic disorders, including infective, auto-immune, inflammatory, or neoplastic disorders [6].

The differential diagnosis of vasculitis is broad, including infectious causes: bacterial (tuberculosis, syphilis, lyme disease, whipple’s disease, brucellosis, cat scratch disease, endophthalmitis, and post-streptococcal syndrome), viral (human T cell lymphoma virus type1, cytomegalovirus, herpes simplex virus, varicella zoster virus, epstein-barr virus, rift valley fever virus, hepatitis, west nile virus, and dengue fever virus) and parasitic (toxoplasmosis, rickettsia, rocky mountain, and spotted fever). Other causes are neurologic disorders, including multiple sclerosis, cerebral microangiopathy, and susac syndrome. Malignancy can also cause retinal vasculitis, including paraneoplastic syndromes, ocular lymphoma, and acute leukemia. Systemic inflammatory diseases are another descent group, including behçet’s disease, sarcoidosis, systemic lupus erythematosus, wegener’s granulomatosis, polyarteritis nodosa, churg-Strauss syndrome, relapsing polychondritis, slögren’s A antigen, rheumatoid arthritis, HLA-B27-associated uveitis, crohn’s disease, postvaccination, dermatomyositis, takayasu’s disease, buerger’s disease, and vogt-koyanagi-harada disease. Another grope is the ocular diseases, including idiopathic retinal vasculitis, aneurysms, neuro retinitis (IRVAN) syndrome, pars planitis, and birdshot chorioretinopathy. When all other causes are excluded, the diagnosis of Eales’ disease can be made [6, 7].

Our patient underwent a full assessment. Infectious and systemic diseases were ruled out; the presentation was not typical of any other disease. The positive tuberculin skin test in a health care worker of Indian descent with ocular vasculitis, with an otherwise normal evaluation, indicated tuberculosis exposure and led to Eales’ disease diagnosis.

Eales’ disease is an idiopathic inflammatory venous occlusive disease, often bilateral, and affects young, healthy adults, mostly males in their second decade. It is characterized by three overlapping stages: perivascular phlebitis (venous vasculitis), peripheral nonperfusion (occlusion), and retinal neovascularization. The disease affects the peripheral retina, and the clinical presentation may be recurrent vitreous hemorrhages due to neovascular proliferation, which is the leading cause of visual loss [2]. The diagnosis remains a clinical diagnosis of exclusion.

Eales’ disease is commonly reported from the Indian subcontinent, with an incidence of one in 200–250 ophthalmic patients, while rare in developed countries [8]. To this day, the etiology of Eales’ disease remains unclear, and it is considered a primary vasculitis of unknown etiology. Since retinal vasculitis and peripheral retinal neovascularization are associated with various systemic and ocular diseases that mimic Eales’ disease in the inflammatory and proliferative phases, it is crucial to distinguish Eales’ disease from other conditions.

Various studies have been done to identify the etiology of Eales’ disease, and multiple theories have been established. While the association of Eales’ disease with multiple systemic diseases was found to be occasional [9], two studies found tubercle bacilli in pathologic specimens [10, 11]. The tubercular etiology is based on observations of active or healed tuberculosis in some patients with primary vasculitis. However, such association was not more than 1.3% in a large clinical study of patients in India with pulmonary tuberculosis [12]. Many authors have suggested that the etiology is hypersensitivity and an allergic reaction to tuberculoprotein, which develops following exposure to tuberculosis. A positive Mantoux reaction was found in up to 90% in some series [13]. Nevertheless, Eales' disease was reported in Mantoux-negative patients [9]. Light microscopic and immunohistochemical studies have shown predominant T-cell involvement in the lymphocytic infiltration of the epiretinal and subretinal membranes of Eales’ disease. Therefore, predominant T cells probably indicate a cell-mediated immune mechanism. Moreover, the acute onset, the response to systemic corticosteroid, and the lymphocytic infiltration of the vitreous suggest an immune-mediated mechanism [2].

While this immune-mediated inflammation is mainly involving the veins (periphlebitis), the inflammation in Eales’ disease may also involve the retinal arteries (periarteritis), with no or little retinal arteries involvement [14]; In 1956, Kimura *et al.* reported three patients with primary vasculitis (Eales’), of which one patient with primary arterioles involvement vasculitis and two in whom the arterioles and venules were equally involved [15]. In our patient, the retinal arterial inflammation and what was initially thought to be CRAO turned out to be an uncommon manifestation of retinal vasculitis. Retinal arterial vasculitis is an inflammatory process that primarily involves the arterial vessel causing vessel wall damage, leading to lumen narrowing secondary to thickening or total occlusion secondary to thrombosis, resulting in retinal ischemia [16]. The intensive arterial inflammation in our patient caused a secondary retinal artery occlusion. This presentation has not been reported before.

The mild CRVO in our patient is secondary to the milder vein inflammation, which induced vascular intimal proliferation disrupting the laminar flow within blood vessels, and predisposing them to thrombus formation [17].

As seen in our patient, even though Eales’ is a bilateral disease, eye involvement is often asymmetric and usually carries a good prognosis. The management depends on the disease stage, including observation for non-active peripheral vasculitis, medical therapy, mainly systemic steroids, and adjuvant periocular steroids; generally, the response to steroids is excellent; therefore, it rarely requires other immunosuppressive drugs, including cyclosporine or azathioprine. Laser photocoagulation is the treatment of choice in the proliferative stage. Pars plana vitrectomy is indicated in cases with persistent vitreous hemorrhage with/without retinal detachment [18].

We reported an unusual Eales’ disease case, presenting mainly as CRAO combined with mild CRVO secondary to primary vasculitis. To our knowledge, this association has yet to be reported. Ruling out other causes of retinal vasculitis is crucial before diagnosing Eales’ disease.

## Data Availability

Not applicable.

## Abbreviations

CRAO: Central retinal artery occlusion
CRVO: Central retinal vein occlusion
FA: Fluorescein angiography
IRVAN: Idiopathic retinal vasculitis, aneurysms, neuro retinitis
VA: Visual acuity
